# Integrating food webs with metabolic networks: modeling contaminant degradation in marine ecosystems

**DOI:** 10.3389/fgene.2015.00020

**Published:** 2015-02-03

**Authors:** Georg Basler, Evangelos Simeonidis

**Affiliations:** ^1^Department of Environmental Protection, Estación Experimental del Zaidín Consejo Superior de Investigaciones CientíficasGranada, Spain; ^2^Luxembourg Centre for Systems Biomedicine, University of LuxembourgEsch-sur-Alzette, Luxembourg; ^3^Institute for Systems BiologySeattle, WA, USA

**Keywords:** flux balance analysis, ecosystem modeling, food webs, biodegradation, PCBs, marine ecosystems, network analysis, genome-scale metabolic modeling

The seas are continuously pervaded by a broad range of contaminants entering the marine environment from polluted soils, the atmosphere, sewage, water transport or river streams (Shahidul Islam and Tanaka, 2004). Organic pollutants are of particular concern, because of their tendency to accumulate in specific organisms, thus threatening both ecosystem stability and human health (Fleming et al., 2006; Johnston and Roberts, 2009). Among those, polychlorinated biphenyls (PCBs) are synthetic compounds produced by the chemical industry and found throughout global environments (Beyer and Biziuk, 2009). Due to their hydrophobicity, PCBs accumulate in the lipids of marine species, leading to bioaccumulation predominantly at higher trophic levels (Perugini et al., 2004). On the other hand, PCBs can be degraded by the sequential anaerobic and aerobic metabolic processes of microorganisms (Borja et al., 2005). Consequently, the accumulation and fate of these pollutants within ecosystems depend not only on their uptake by and transfer among marine species, but also on microbial biodegradation. The biostimulation of degrading bacteria or their insertion (bioaugmentation) at contaminated sites may represent a promising strategy for bioremediation (Tyagi et al., 2011).

Biomass transfer among species of a food web can be modeled using allometric scaling rules describing body size, consumption and metabolism (Yodzis and Innes, 1992; Berlow et al., 2009). Similarly, the flow of contaminants through a food web can be estimated by mass-balance modeling of chemical transfer and adsorption (Wania and Mackay, 1999; Arnot and Gobas, 2004; Breivik et al., 2007; Nichols et al., 2009; Taffi et al., in press). Such modeling approaches can be complemented by measurements of contaminant concentrations (Kelly et al., 2007). Recently, a food web representing the major ecological groups of the Adriatic Sea and their predator-prey interactions (Coll et al., 2007) was integrated with PCB concentration data obtained from an extensive literature review (Taffi et al., in press). The resulting model allowed prediction of the flow rates of PCBs among ecological groups and reproduced the finding that PCBs accumulate mostly in species at higher trophic levels and with lower total biomass. Further, the model allowed estimation of the fate of contaminants, such as their bioaccumulation in marine species.

The described efforts facilitated generic estimations of contaminant flows in ecosystems, but largely neglected the contribution of microorganisms to pollutant degradation. Specifically, PCBs can be dechlorinated and degraded by a range of bacteria (Borja et al., 2005). Thus, biodegradation may be an important factor for the persistence of a pollutant within an ecosystem and, moreover, may provide hints for bioremediation strategies based on the aforementioned bacteria. The uptake, degradation and excretion of compounds by microorganisms can be predicted from the knowledge of their metabolic capabilities using constraint-based approaches (Bordbar et al., 2014). Today, detailed metabolic reconstructions are available for a broad range of organisms (Monk et al., 2014), including several bacteria known for their bioremediation capabilities, such as *Geobacter* spp. (Mahadevan et al., 2011), *Shewanella oneidensis* (Fredrickson et al., 2008), and *Pseudomonas putida* (Nogales et al., 2008); and new reconstructions for more organisms are published regularly. Thus, an intriguing challenge is the combination of ecological and metabolic modeling approaches to integrate population-level simulations of ecosystems with cellular modeling of microbial metabolism, similar to the previous integration of reactive transport models with metabolic models (Fang et al., 2011).

Taffi et al. (2014) have recently integrated a comprehensive food web of the Adriatic Sea with PCB concentration data and a metabolic model of *P. putida*. First, the authors conducted an extensive literature review to complement a food web reconstruction (Coll et al., 2007) with data of PCB concentrations among marine species (Taffi et al., in press). Next, linear inverse modeling was used to infer unknown contaminant flows and concentrations based on the mass balance principle. Finally, the ecological network was integrated with the genome-scale metabolic network of *P. putida* for constraint-based simulation of microbial PCB degradation. The study integrated two methodologically similar modeling approaches within a reaction-based ecological/microbial network representation, relying on the parallels between representing PCB concentrations and flows on the ecosystem level, and metabolite concentrations and reaction fluxes on the microbial cellular level. Therein, marine species groups resemble the representation of metabolites, while contaminant flows are modeled as reactions. This approach enabled the seamless integration of ecological and metabolic modeling techniques, providing the basis for multi-scale simulations of ecosystems.

The modeling approach was used to predict the influence of different microbial bioremediation strategies on the fate and distribution of PCBs in marine species of the Adriatic Sea. The effect of varying oxygen levels on microbial PCB degradation revealed a tradeoff between PCB uptake and growth of *P. putida*. Further, the impact of different bioremediation scenarios on global and local network indices was assessed. Importantly, the generality of the proposed approach facilitates the integration of measured data, the incorporation of established techniques from ecological and metabolic modeling and the direct application of the methodology to other ecological and microbial networks. Thus, it can be used to guide the selection of appropriate bacteria and consortia (Thompson et al., 2005), or the design of genetically engineered bacteria for bioremediation (Singh et al., 2011). The approach will stimulate new developments in ecological modeling and offer insights into the multi-level interplay among ecosystems, microbial networks and biodegradation.

## Conflict of interest statement

The authors declare that the research was conducted in the absence of any commercial or financial relationships that could be construed as a potential conflict of interest.
